# *Arabidopsis* serine/threonine/tyrosine protein kinase phosphorylates oil body proteins that regulate oil content in the seeds

**DOI:** 10.1038/s41598-018-19311-3

**Published:** 2018-01-18

**Authors:** Iyappan Ramachandiran, Anitha Vijayakumar, Visvanathan Ramya, Ram Rajasekharan

**Affiliations:** 1 Department of Biochemistry, Indian Institute of Science, Bangalore, 560012 India; 2 Lipidomics Center, Central Food Technological Research Institute, Mysore, Karnataka 570020 India

## Abstract

Protein phosphorylation is an important post-translational modification that can regulate the protein function. The current knowledge on the phosphorylation status of plant oil body (OB) proteins is inadequate. This present study identifies the distinct physiological substrates of *Arabidopsis* serine/threonine/tyrosine protein kinase **(**STYK) and its role in seed oil accumulation; the role of *Arabidopsis* OLE1, a major seed OB protein has also been elucidated. *In vitro* kinase assay followed by mass spectrometry identifies residue that are phosphorylated by STYK. Further, co-expression of OLE1 and STYK in yeast cells increases the cellular lipid levels and reduces the total lipid when OLE1 was replaced with OLE1^T166A^. Moreover, i*n vivo* experiments with OB isolated from wild-type and *styk* knock-out lines show the ability of STYK to phosphorylate distinct OB proteins. OLE1^T166A^ mutant and *Arabidopsis styk* mutant demonstrate the significant reduction of its substrate phosphorylation. *styk* mutant line significantly reduces the amount of total seed oil as compared to wild-type seeds. Together, our results provide the evidences that Arabidopsis At2G24360 (STYK) is phosphorylating oil body proteins and the phosphorylation regulates the oil content in Arabidopsis seeds.

## Introduction

In plants, oil body (OB) proteins are highly conserved and are grouped into structural proteins or enzymes^1^. Seed OBs contain a variable number of proteins, depending on the oleaginous plants and their tissues^2,3^. The major seed OB proteins oleosin, caleosin and steroleosin have been shown to play an important role in regulating the OB structure and lipid accumulation^4^. There are other minor proteins in OBs that are reported to be involved in solute transport, protein synthesis and vesicular transport^3^. The proteomics data for the purified seed OBs from more than 18 species, including nine crop plants provide a detailed insight into the nature of the proteins belonging to the organelle and the diversity of OB functions^1^. However, there has been no dedicated study to report the different phosphoproteins of the seed OBs.

Neutral lipids that are stored in the OBs break down following seed imbibition and germination to provide energy and carbon skeletons to support the growth of seedling^5,6^. Recent studies have indicated that post-translational modifications (PTM) of OB proteins play a role in these interactions. Specifically, the ubiquitinylation of oleosin and caleosin in germinating sesame seeds has been proposed to play a role in the interaction of OBs with glyoxysomes which is needed for breaking down the lipids to release free fatty acids to provide energy^7^. Similarly, the phosphorylation of oleosin has been suggested to help in the interaction of OBs with lipases and other enzymes of lipogenic pathway^5^. Previously, we have shown that peanut oleosin 3, although a structural protein, acts as a bifunctional enzyme with both monoacylglycerol acyltransferase and phospholipase activities^8^. Furthermore, a recent report has demonstrated the role of the proteasome in the degradation of ubiquitinylated oleosins^9^; oleosin degradation is required for lipid mobilization which further highlights the role of PTM in regulating the function of seed OB proteins.

The current knowledge on the phosphorylation status of plant seed OB proteins is vastly inadequate. There is only one report showing the phosphorylation of steroleosin^10^. Although there are many *in silico* phosphorylation sites predicted for caleosin, there are only two reports showing its partial phosphorylation^11^. OLE5 and OLE2 from *Arabidopsis thaliana* have been recently shown to be phosphorylated during lipid droplet degradation^9^. However, the protein kinase phosphorylating these proteins has not been reported. Previous studies from our laboratory have identified a non-mitogen activated dual specificity STY protein kinase from *Arachis hypogaea* (AhSTYK) that is involved in cold, salt stress and seed development. The transcript of AhSTYK was also shown to be increased in the mid-cotyledonary stage of seed development indicating the possibility that AhSTYK may be involved in signal transduction mechanisms related to storage of metabolites^12,13^. Furthermore, our earlier studies have also identified a Mn^2+^ dependent dual-specificity kinase (AtSTYK), a homolog of AhSTYK in *Arabidopsis*^14,15^. However, the physiological substrate for these protein kinases has not been identified despite its physiological significance in abiotic stress and seed development. Interestingly, we have recently shown that AhOLE3 is phosphorylated by AhSTYK *in vitro* and that the phosphorylation regulates the bifunctional activity of AhOLE3^16^. Nonetheless, the *in vivo* phosphorylation of AhOLE3 was not shown. These studies demonstrate the current need for the identification of phosphorylated proteins and their corresponding kinases in plant seed OBs of Arabidopsis.

The present study is directed to identify the role of *Arabidopsis* phosphorylated OB proteins in seeds and their corresponding protein kinases. Using a combination of radiometric and proteomic approaches, we have identified the different phosphorylated OB proteins from *Arabidopsis* seeds. *Arabidopsis* has fifty-seven distinct STY protein kinases^17^. However, both our *in vitro* experiments in yeast and *in vivo* experiments in yeast and Arabidopsis demonstrated that the AtSTYK (AT2G24360) is one of the major protein kinases phosphorylating these OB proteins and which in turn affects the seed oil content. Seed OBs contain a variable number of proteins depending on the oleaginous plants and their tissues. Oleosins are the most abundant OB proteins that control OB structure and lipid accumulation^18^. Although, we show various OB protein phosphorylation, the role of OLE1 in regulating the lipid content and its effect upon phosphorylation has been demonstrated in detail.

## Results

### STYK phosphorylates OLE1 in vitro

**Figure 1:**
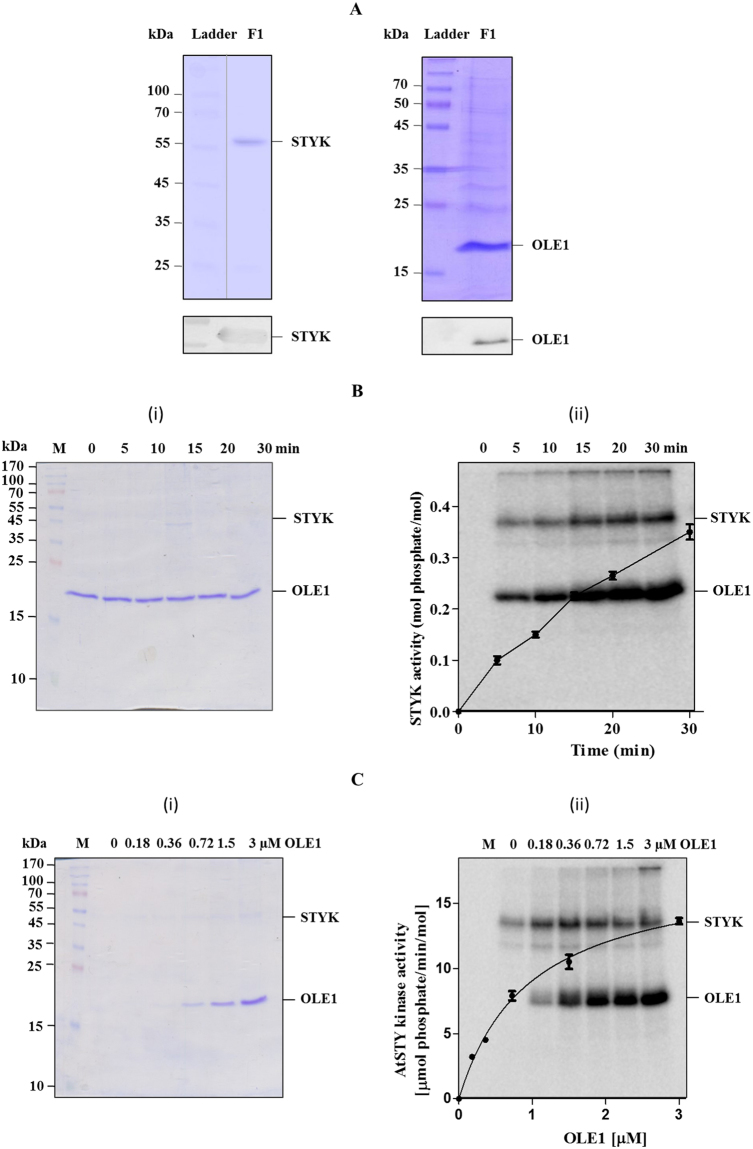
STY protein kinase phosphorylates OLE1. (**A**) SDS-PAGE profile of bacterially expressed, affinity column purified STYK and OLE1 (left and right panel respectively). Lower panels, confirmation of STYK and OLE1 proteins using anti-STYK and anti-His_6_ monoclonal antibody respectively. *In vitro* phosphorylation of the purified, bacterially expressed OLE1 by STYK. (**B**) time- and **C**, protein-dependent phosphorylation. After the reaction, the mixture was resolved on a 15% SDS-PAGE and stained with (i) Coomassie brilliant blue followed by (ii) phosphorimaging. Inset shows the radioactivity quantification from bands excised from gels determined by liquid scintillation counting. Data represent mean ( ± SD) of three independent experiments. F1, purified protein fraction.

To identify the substrate for *Arabidopsis* STYK, a standard *in vitro* kinase assay was performed using *E. coli*-expressed and purified GST-STYK (Fig. 1A, left panel, Supplementary fig. S4A) and *E. coli*-expressed and purified His_6_-OLE1 (Fig. 1A, right panel, Supplementary fig. S4B). Phosphorimaging analyses of the SDS-PAGE-separated kinase assay product showed that OLE1 is indeed a substrate for STYK. The STYK activity depended on the time of the reaction (Fig. 1B), the amount of OLE1 that was used (Fig. 1C) and the amount of ATP that was used in the reaction (Supplementary Fig. S3A).

### OLE1 is mono-phosphorylated at a threonine residue

**Figure 2:**
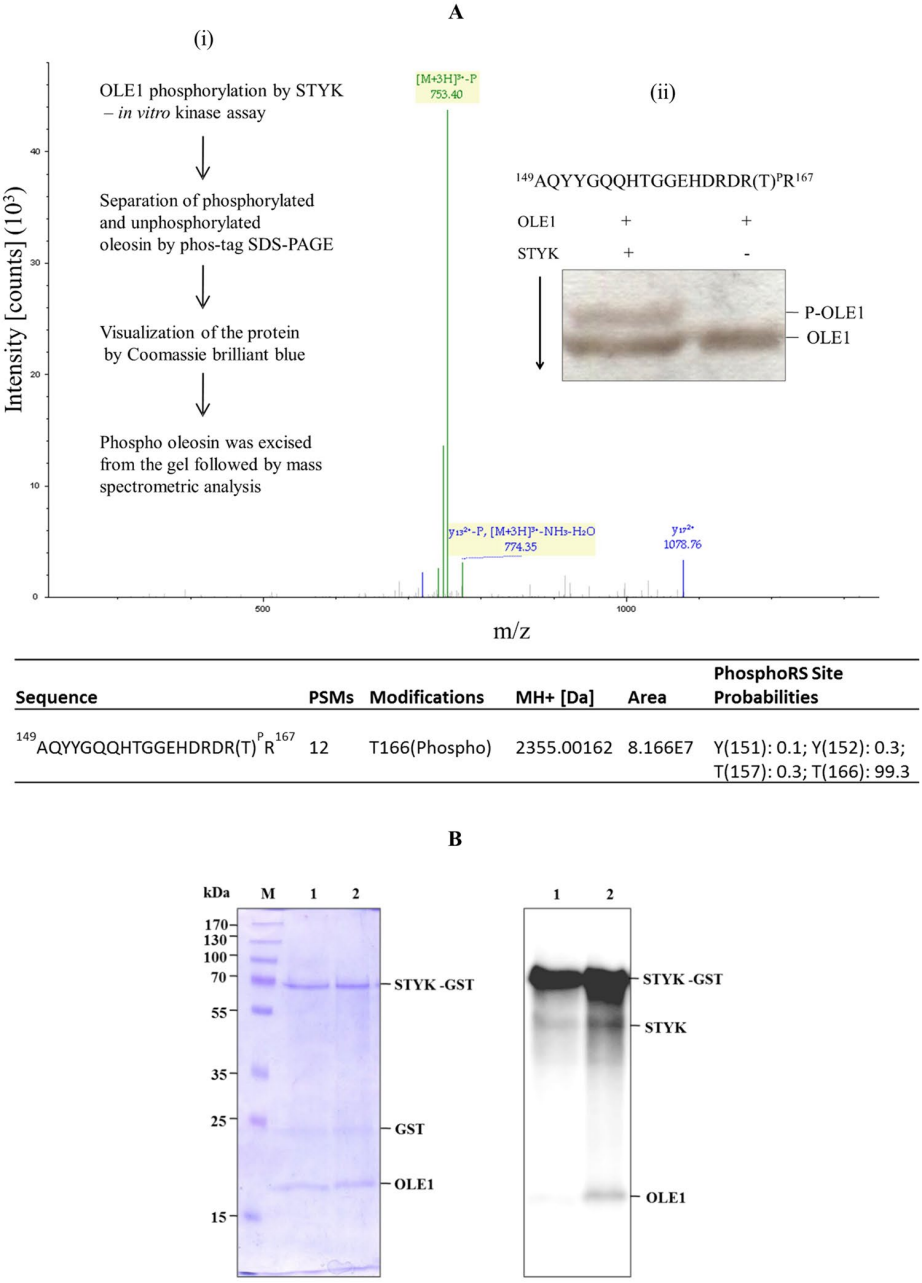
Identification of OLE1 phosphorylation site. (**A**) LC-MS/MS spectra of a phosphorylated peptide from OLE1. The inset (i) shows a brief procedure. The inset (ii) shows the sequence of the phosphopeptide identified and the separation of a phosphorylated-OLE1 from unphosphorylated OLE1 by Mn^2+^–Phos-tag SDS-PAGE stained by silver nitrate. Lower table shows the phosphorylation site identification by mass spectrometry. (**B**) site-directed mutagenesis of the phosphorylation site of OLE1. The kinase assay was performed in a reaction mixture of 1 μg of STY kinase, 1 μg of OLE1 or T166A mutant, 0.5 μCi of [^32^P]ATP, 25 μM ATP, and 10 mM MnCl_2_. The reaction was carried out at 30 °C for 30 min. Phosphoproteins were resolved on a 12% SDS-PAGE and stained with Coomassie brilliant blue (i) followed by phosphorimaging (ii) Lane 1, OLE1^T166A^ mutant; lane 2, WT-OLE1. PSMs, Peptide spectral match. **↓**, in the inset (ii) indicates the direction of electrophoretic migration.

An *in vitro* experiment suggested the OLE1 phosphorylation by STYK. Further, experiments were conducted to identify the phosphorylation site of OLE1. The phosphorylated and unphosphorylated OLE1 were separated on a SDS-PAGE gel containing phos-tag acrylamide (Fig. 2A ii, Supplementary fig. S4C). The phosphorylated OLE1 was digested with trypsin, and the peptide mixture was analyzed by mass spectrometry. Liquid chromatography-tandem mass spectrometry analysis of the tryptic digests confirmed the presence of OLE1 with good sequence coverage. In addition, the mass spectrometric analyses of the OLE1 peptide samples revealed the presence of a single phosphopeptide with a Thr-166 phosphorylation, represented by the peptide AQYYGQQHTGGEHDRDR(T)^P^R (Fig. 2A). Furthermore, to validate whether the Thr-166 residue of OLE1 is the phosphorylation site of STYK, site-directed mutagenesis accompanied by an *in vitro* kinase assay was performed. The putative phosphorylation residue of the OLE1 protein was replaced by an alanine ‘A’ residue. Plasmid constructs were generated to encode the mutated recombinant OLE1 protein (OLE1^T166A^). The mutant protein was expressed in *E. coli* and the purified protein was subjected to *in vitro* kinase assay. Interestingly, the phosphorylation of the mutant protein OLE1^T166A^ by STYK was completely abolished (Fig. 2B). These results indicate that Thr-166 amino acid is indeed important for the phosphorylation OLE1 by STYK.

### Role of recombinant oleosin and STYK in yeast cells

**Figure 3:**
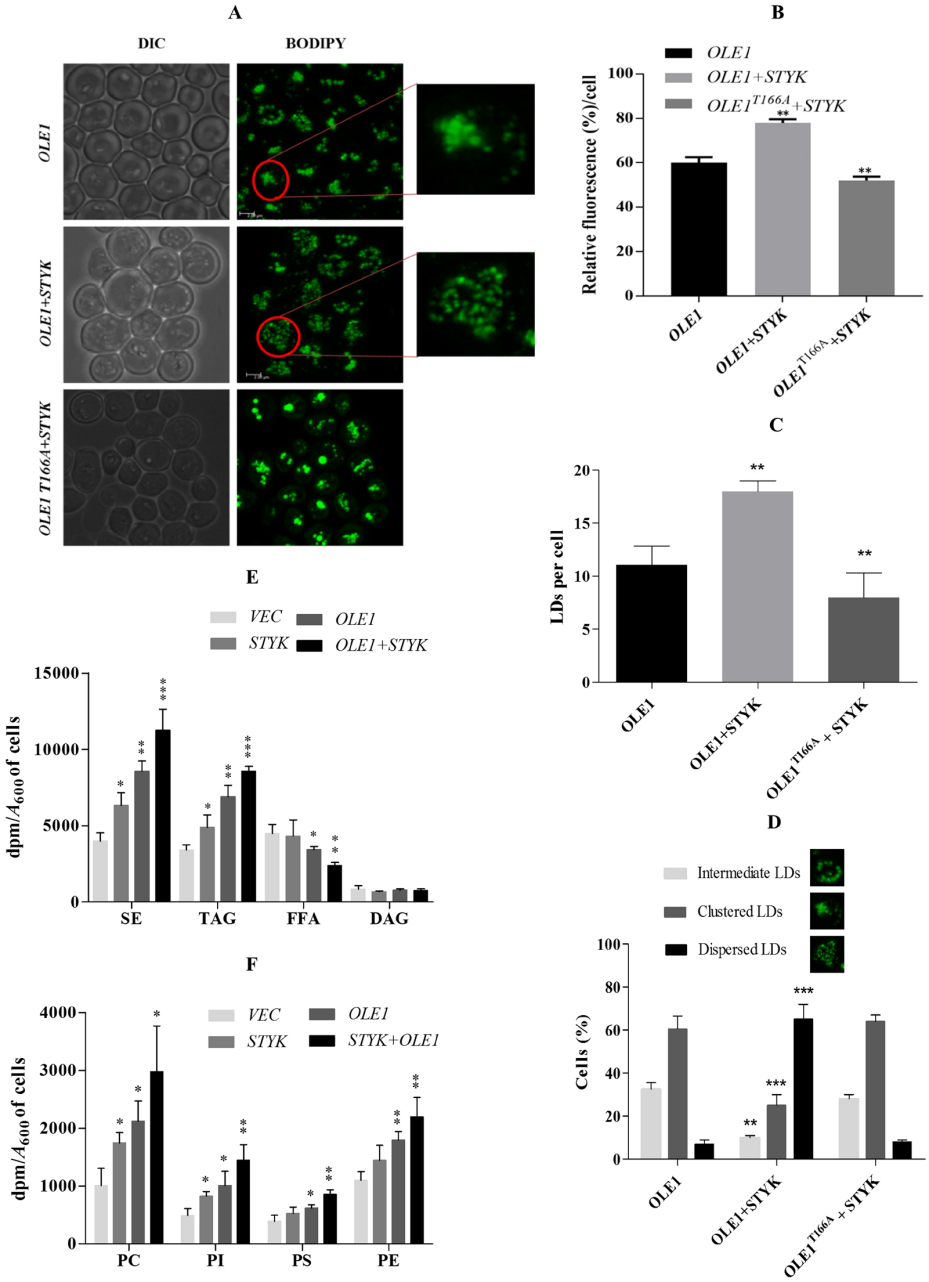
*In vivo* function of OLE1 in *S. cerevisiae*. (**A**) BODIPY493/503-stained lipid droplets were observed using confocal microscopy. DIC, Differential interference contrast. (**B**) quantification of fluorescense (**C)**, quantification of the number of lipid droplets (**D)**, quantification of the changes in lipid droplet morphology The data presented represents means ( ± SD) of one twenty five cells of three biological replicates (3 × 125 cells). **E**, [^14^C]acetate incorporation into TAG, SE and FFA was quantified. The vector control, *OLE1-, STYK-* and *OLE1 + STYK*-transformed yeast cells were grown to stationary phase in the presence of galactose and 0.2 µCi of [^14^C]acetate/mL culture medium. The cells (*A*_600_ = 25) were lysed using glass beads. Lipids were extracted and resolved on a silica-TLC plate using petroleum ether/diethyl ether/acetic acid (70:30:1, v/v) as the solvent system. **F**, phospholipids profiling. The extracted total lipids were run on a chloroform/methanol/ammonia (65:25:5, v/v) solvent system as the first dimension followed by a chloroform/methanol/acetone/acetic acid/water (50:10:20:15:5, v/v) solvent system as the second dimension. Error bars indicate SD (n = 4). Asterisks indicate significant differences compared to the vector controls (*P < 0.05 and **P < 0.01). SE, steryl esters, TAG, Triacylglycerol, FFA, Free fatty acid, DAG, Diacylglycerol, PC, Phosphatidylcholine, PI, phosphatidylinositol, PE, Phosphatidylethanolamine, PS, Phosphatidylserine.

The effect of phospo-OLE1 in cellular function was studied by co-expressing OLE1 and STYK in yeast cells (W3031-A). The lipid droplets (LD) of yeast cells are structurally and functionally related to those of plant and animal cells. Hence, yeast is an ideal model to study the lipid droplet associated function of these proteins. To study the effect of phospho-OLE1 on its cellular function, both STYK and OLE1 were overexpressed as an N-terminal His_6_-tagged protein in *Saccharomyces cerevisiae* under a constitutive GPD promoter and GAL promoter respectively. The BODIPY493/503 staining of the LDs of yeast cells overexpressing OLE1 and or STYK showed alteration in the lipid droplets levels (Fig. 3A). Further, quantification of fluorescence also confirms the visual examination of an increase and decrease in lipid droplets (Fig. 3B). Interestingly, the number of LDs were increased significantly upon OLE1 and STYK co-expression in yeast cells as compared to OLE1 alone (Fig. 3C). On the other hand, decrease in LDs was also observed when STYK was co-expressed with OLE1^T166A^ mutant. It was also noted that the pattern of LDs was altered when kinase is overexpressed (Fig. 3D). Further, to study the lipid accumulation in yeast cells, the influence of STYK phosphorylation of OLE1 on TAG accumulation was examined by overexpressing *STYK*, *OLE1* and *STYK* + *OLE1* in yeast cells in the presence of [^14^C]acetate followed by lipid analysis. Metabolic labeling studies showed that the level of TAG increased in the cells overexpressing different gene constructs compared to the vector control on the order of STYK + OLE1 > OLE1 > STYK (Fig. 3E). The phospholipids levels and the total cellular fatty acids level were also significantly increased in yeast cells overexpressing different gene constructs (Fig. 3F, Supplementary Fig. S6). Taken together, the above experiments clearly demonstrated that *Arabidopsis* STYK is capable of phosphorylating OLE1 in yeast cells and there by altering the cellular lipid levels when expressed in a heterologous manner.

### Arabidopsis seed oil body proteins are phosphorylated in vivo

**Figure 4:**
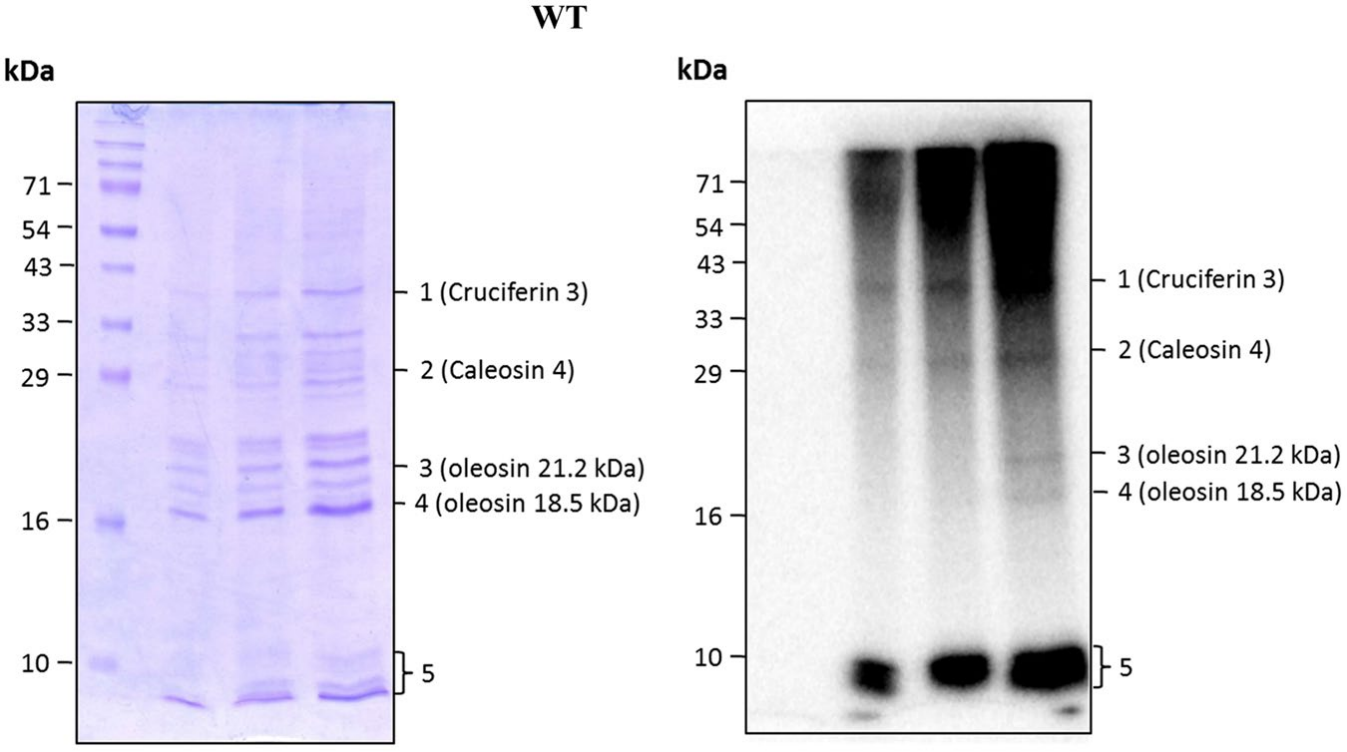
*In vivo* phosphorylation of *Arabidopsis* OB proteins. Col-0 seeds were imbibed in the presence of 250 µCi of [^32^P]orthophosphoric acid for 36 h (the radicles emerged). The seeds were ground, and the OBs were isolated and purified by sucrose density gradient centrifugation. The purified OBs were delipidated using diethyl ether, and the proteins were resolved on a 15% SDS-PAGE. Coomassie brilliant blue staining of proteins (left). Phosphorimaging of the same gel (right).

To validate the *in vitro* phosphorylation and to check the phosphorylation status of OLE1 *in vivo*, experiments were conducted using *A. thaliana* seed oil body (OB) proteins. The phosphorylation status of *Arabidopsis* OB proteins was investigated using [^32^P]orthophosphoric acid-imbibed seeds. The isolated OB proteins from the radio-labeled seeds were resolved onto a 15% SDS-PAGE gel and subjected to phosphorimaging analysis. Interestingly, the labeling experiment revealed approximately five distinct proteins in the range of 5–70 kDa (Fig. 4, Supplementary Fig. S5). The distinctly separated protein bands were excised from the gel, and their identity was elucidated using LC–MS/MS analysis. The proteins that were identified using mass spectrometric analyses are listed in Table 1. The mass spectrometric analyses revealed that the identified proteins were caleosin 4, oleosin 2, and oleosin 1 (bands 2, 3 and 4, respectively). Band 1 was identified as a 12 S seed storage protein, cruciferin 3 (CRU3). 12 S seed storage proteins are the most abundant and phosphorylated proteins in *Arabidopsis* seeds. A number of proteins were detected in the band containing the clustered proteins (band 5). The major identified proteins were ubiquitin and polyubiquitin fragments. Furthermore, the proteins with high MASCOT scores and sequence coverage that were identified in the clustered band are listed in Table 1.

**Table 1:** Identification of phospho-OB proteins by LC-MS/MS.

Band No	Accession	Protein name	(%) Coverage	Unique Peptides	PSMs	AA	MW [kDa]	Gene
1	75251070	Cruciferin 3	55.73	20	108	524	58.19	At4g28520
2	Q9CAB7	Probable peroxygenase 4; or caleosin 4	69.23	20	199	195	22.1	At1g70670
3	Q39165	Oleosin 21.2 kDa	33.17	7	39	199	21.27	At5g40420
4	P29525	Oleosin 18.5 kDa	35.26	7	25	173	18.56	At4g25140
5	Q94K78	Putative uncharacterized protein	42.11	2	2	57	5.9	At2g40765
5	Q9XIA7	Mitochondrial import receptor subunit TOM6 homolog	38.89	2	2	54	6.26	At1g49410
5	Q0WRN6	Polyubiquitin 4 UBQ4	35.61	0	2	351	39.25	At5g20620
5	A6XI99	Ubiquitin (Fragment)	34.25	0	2	219	24.55	At4g05320
5	P15459	2 S seed storage protein 3	24.39	5	27	164	18.7	At4g27160

Phosphorylated bands were excised and subjected to in-gel trypsin digestion. The digested peptides were reconstituted in 15 μL of 0.1% formic acid and 3 μL of the same was injected onto a column. The digested peptides were subjected to a standard 70 min RPLC-MS/MS analysis with collision-induced dissociation as the fragmentation method. The generated data were searched following the standard approach using MASCOT 2.4 as a search engine on Proteome discoverer 1.4. The data were searched against the UniProt Swiss-Prot database (non-redundant database with reviewed proteins), UniProtTrEMBL database (database with unreviewed proteins) and *Arabidopsis* database downloaded from NCBI. A minimum of two highly confident peptides were used as a prerequisite to identify the proteins. PSMs, Peptide spectral match.

### STYK phosphorylates CAL4 at multiple residues

From the mass spectrometry data, it was suggested that phosphorylation was also observed with other oil body proteins. Among the OB proteins apart from oleosins, the phosphorylation of caleosin was significantly high in wild-type OB proteins. To validate whether the CAL4 that was identified by the proteomic approach as described earlier is also a substrate for STYK, an *in vitro* kinase assay was performed using Ni-NTA affinity purified CAL4 (Supplementary Fig. S2A) and STYK. Phosphorimaging analyses of the SDS-PAGE-separated kinase assay product confirmed that CAL4 is indeed a substrate for STYK. The STYK activity was dependent on the time of the reaction (Supplementary Fig. S2B), the amount of CAL4 that was used (Supplementary Fig. S2C) and the amount of ATP that was used in the reaction (Supplementary Fig. S3B). Furthermore, analyses of CAL4 peptide samples following an *in vitro* kinase assay revealed the presence of multiple phosphopeptides, enabling the assignment of at least three phosphorylation sites. A comparison of the mass spectrometry peak areas of the CAL4 phosphopeptides indicated that Ser-177, represented by the peptide AAYDG(S)^P^LFEKLEK, is the most phosphorylated site, followed by Thr-39, represented by the peptide NKDGIVYPSE(T)^P^FQGFR, and Ser-75, represented by the peptide GF(S)^P^IWFPIEVK (Supplementary Table S1).

### Phosphorylation of OLE1 and CAL4 by STYK is specific

**Figure 5:**
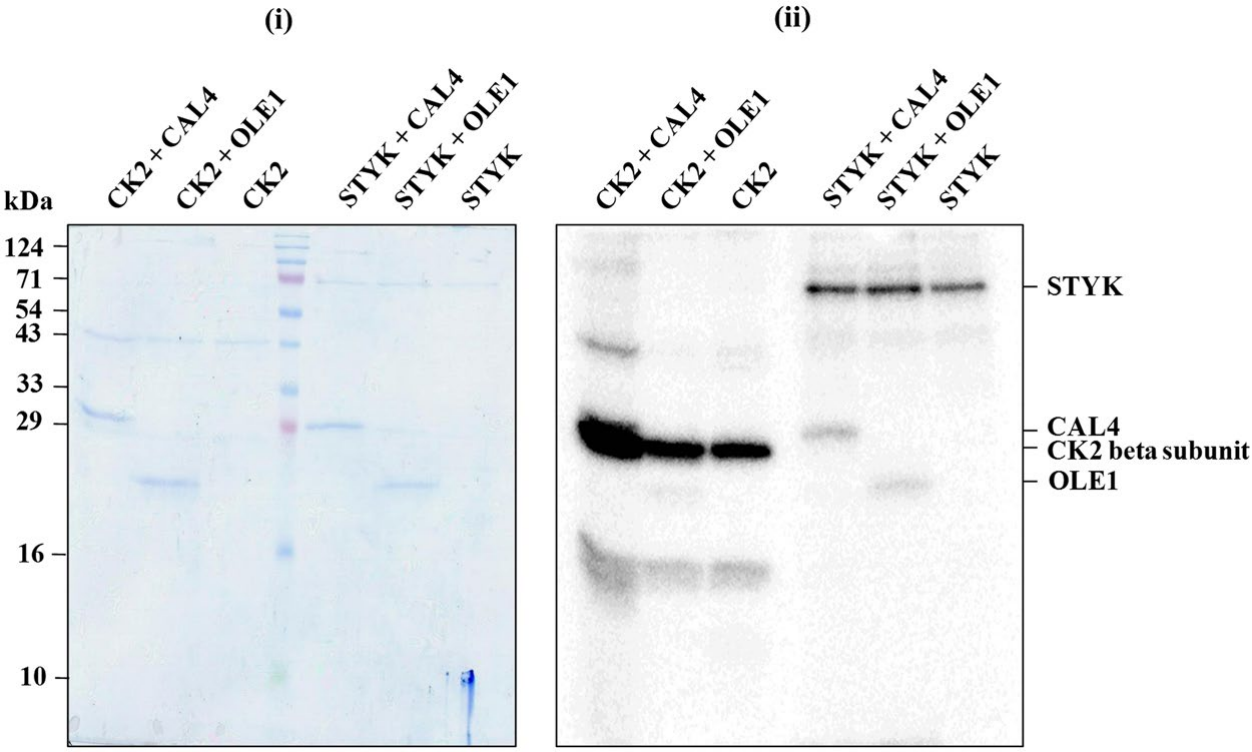
STYK phosphorylates OLE1 and CAL4 specifically*. In vitro* kinase assay was performed using the purified, bacterially expressed OLE1 and CAL4 by casein kinase 2 (CK2) and AtSTY protein kinase. The reaction mixture was resolved on a 15% SDS-PAGE and stained with (i) Coomassie brilliant blue followed by (ii) phosphorimaging.

To determine whether the OB proteins OLE1 and CAL4 are phosphorylated specifically by STYK and are not a generic substrate for protein kinases, we performed an *in vitro* kinase assay using a control kinase, casein kinase 2 (CK2). CK2 has been shown to phosphorylate lipid droplet proteins in yeast^19^. STYK was used as a source of protein kinase in the control reaction. Phosphorimaging analyses of the SDS-PAGE-separated kinase assay product showed that OLE1 and CAL4 were significantly phosphorylated by STYK (Fig. 5). A very faint signal of OLE1 was seen in the lane containing CK2. Both STYK and beta subunit of CK2 were autophosphorylated. However, the CK2 autophosphorylation in the presence of CAL4 was quite prominent. A significantly increased substrate phosphorylation of the OB proteins OLE1 and CAL4 by STYK further prove that STYK specifically phosphorylates both the OB proteins and that OLE1 and CAL4 are not generic substrates for protein kinases.

### STYK (At*2*g24360) phosphorylates Arabidopsis OB proteins

**Figure 6:**
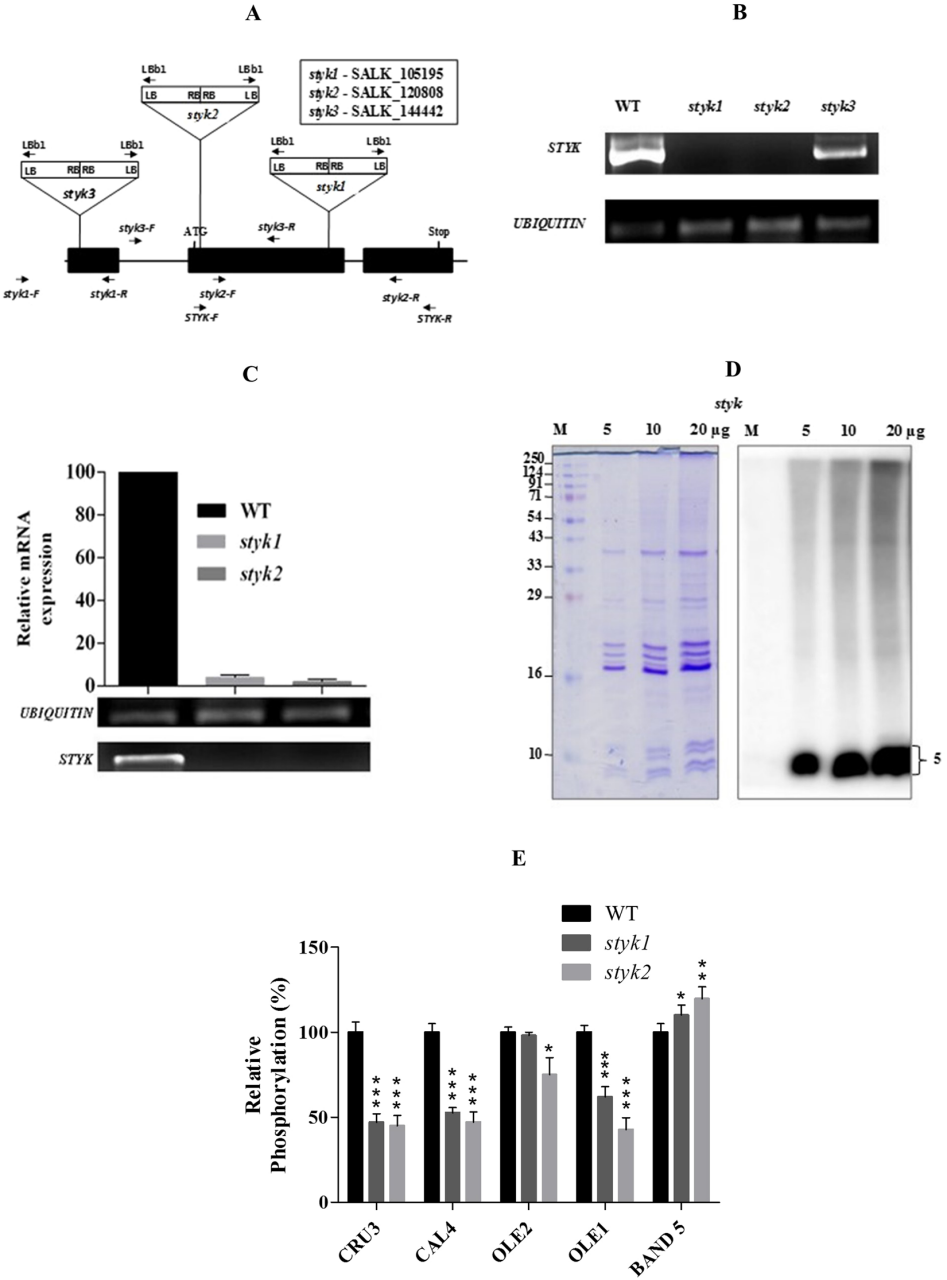
Characterization of *Arabidopsis styk* knock-out plants for *in vivo* phosphorylation. (**A)**, schematic representation of the site of T-DNA insertion on STYK genomic DNA of various germplasms. (**B**) RT-PCR confirmation of *styk* knock-out lines for T-DNA insertion using cDNA synthesized from equal amount of RNA (3 µg). (**C**) qRT-PCR confirmation transcript levels of *STYK* using gene specific primer. Ubiquitin was used as an internal control for all the PCR experiments. (**D**) representative figure of *in vivo* OB protein phosphorylation in *styk* mutant seeds. *styk* mutant seeds were imbibed in the presence of 250 µCi of [^32^P]orthophosphoric acid for 36 h (the radicles emerged). The purified OBs were delipidated using diethyl ether, and the proteins were resolved on a 15% SDS-PAGE. *Left panel*, Coomassie brilliant blue staining of proteins. *Right panel*, phosphorimage of the same gel. **E**, the radiolabel associated with the different protein bands of WT and *styk* OB proteins were quantified using Multi Gauge V3.0 software (Fuji Film). Data represent mean ( ± SD) of three independent experiments. Asterisks indicate significant differences compared to the Col-0 controls (*P < 0.05, **P < 0.01, ***P < 0.001).

*Arabidopsis* has fifty-seven distinct STY protein kinases^17^. To test whether STYK (AT2g24360) is responsible for the phosphorylation of OB proteins in *Arabidopsis*, T-DNA insertion mutants of *STYK* were obtained from ABRC. The site of T-DNA insertions in *styk1* (SALK_105195), *styk2* (SALK_120808), and *styk3* (SALK_144442) mutants was given in the pictorial representation (Fig. 6A). Further, the true STYK knock-out mutants were screened by PCR with T-DNA border specific primers followed by PCR with gene specific primer. Both RT-PCR and qRT-PCR data showed no transcript levels in *styk1* and *styk2* confirming that only those two lines are the true knock out mutants whereas in *styk3* the presence of transcript was found (Fig. 6B and C). To validate the phosphorylation of OB proteins by STYK, attempts were made to isolate OBs from *styk1* and *2* knock-out lines. The OBs were purified from the [^32^P]orthophosphoric acid-imbibed seeds of the *styk* mutants and analyzed by SDS-PAGE and phosphorimaging. These analyses revealed that OB proteins ranging from 5–10 kDa were phosphorylated prominently in all lanes, like that of the band 5 observed in wild-type OB proteins. However, the phosphorylation of bands 1 to 4 was significantly reduced in the *styk* mutants, indicating that AtSTYK would be the possible kinase that is responsible for the phosphorylation of OB proteins (Fig. 6D, Supplementary Fig. S5).

To further confirm our hypothesis that At2g24360 is the kinase majorly responsible for OB protein phosphorylation, we examined the expression levels of other STYK genes in *Arabidopsis* by whole genome microarray followed by real-time PCR. Both the gene expression profiling revealed the differentially expressed other Arabidopsis STYK genes. Although the real-time PCR data showed many of the STYK are up/down regulated, none of them seem to have complemented the STYK protein kinase function (Supplementary Fig. 1A and B). These data suggested that abolishment of STYK (At2G24360) activity significantly affected the phosphorylation of seed oil body proteins.

### In planta function of OLE1

**Figure 7:**
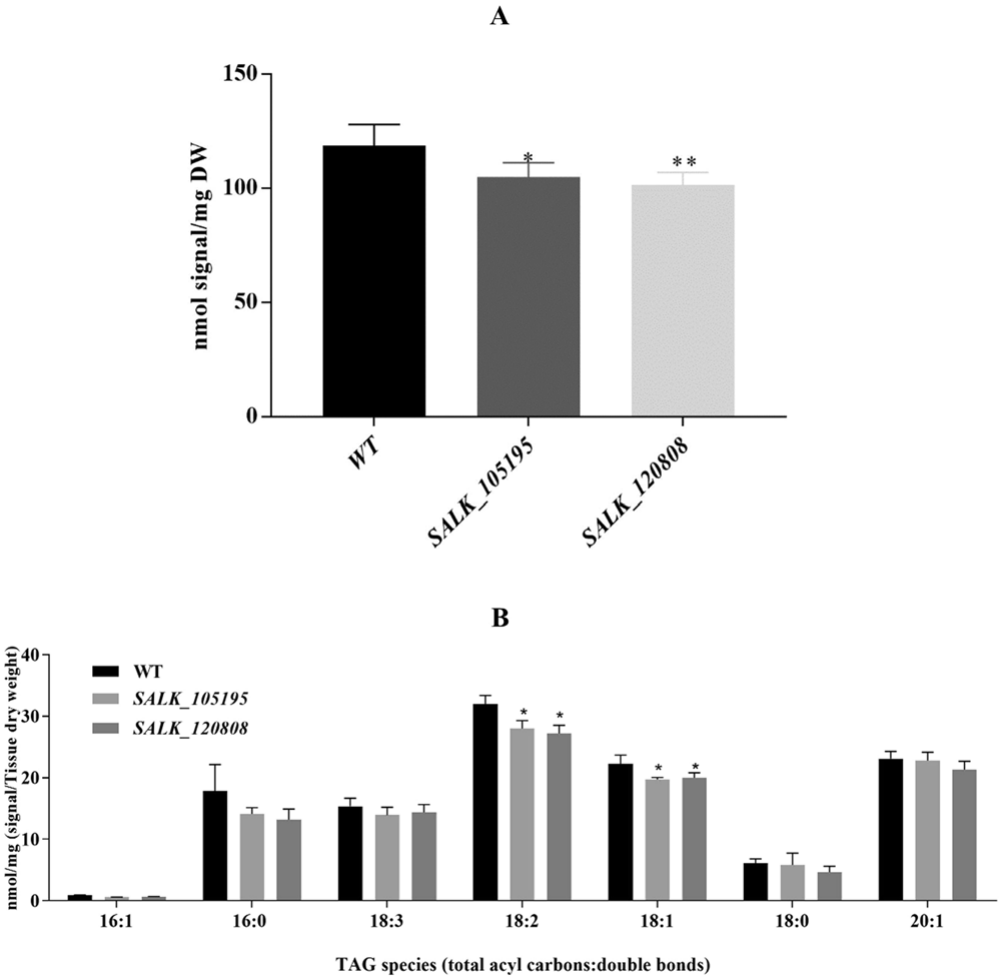
Role of OLE1 phosphorylation in plants. (**A)** total TAG and (**B)**, its corresponding fatty acid molecular species of wild-type and the *styk* knock-out mutant seeds were analyzed using ESI-MS. Error bars indicate SD (n = 4). Asterisks indicate significant differences compared to the WT controls (*P < 0.05 and **P < 0.01).

Further to examine the role of STYK mediated phosphorylation in TAG accumulation *in planta*, we measured the levels of TAG in *styk1* and 2 mutant seeds using mass spectrometry. Interestingly, we found that the seeds of the *styk* mutants had nearly 10–12% less TAG compared to the wild-type seeds (Fig. 7A). Analyses of TAG molecular species revealed a significant reduction in both unsaturated fatty acids (16:1, 18:2) and saturated fatty acids (18:0) containing TAG compared to wild-type seeds (Fig. 7B). These data suggest that phosphorylation of OLE1 by STYK may regulate the seed TAG levels.

## Discussion

To date, oil body proteome from many plant sources have identified oleosins, caleosins, and steroleosins as the major oil body proteins. A number of minor proteins/enzymes that are involved in making, modifying, and degrading TAG, phospholipids, and sterols have also been identified in these studies^20,21^. However, in many studies, the precise connection between many kinases and the identified protein phosphorylation has not been elucidated.

An earlier study from our laboratory demonstrated that *Arachis hypogaea* OLE3, a major OB protein, is phosphorylated, and this phosphorylation regulates the bifunctional activity of OLE3^8^. We further identified AhSTYK as the kinase phosphorylating AhOLE3 by an affinity purification method. An *in vitro* kinase assay followed by the mass spectrometric identification of the phosphorylated immuno-pulled proteins identified AhOLE3 as the substrate of AhSTYK^16^.

In this present study, we hypothesized that AtSTYK, the homolog of AhSTYK, could phosphorylate the OB proteins of *Arabidopsis in vivo*. To test this, we used both *in vitro* and *in vivo* approaches. An initial, *in vitro* kinase assays using the purified recombinant OLE1/CAL4 and STYK demonstrated the phosphorylation of OLE1/CAL4 by STYK. AtOLE1 is the closest homolog of AhOLE3 with the percent identity of 32% as compared to AtOLE3 (29%). Specifically, the *Km* values of STYK for OLE1 and CAL4 were 0.9659 µM and 0.9972 µM, respectively, demonstrating that STYK prefers OLE1 and CAL4 equally as substrates, although OLE1 has a slightly greater affinity for STYK. Furthermore, OLE1 is specifically phosphorylated at Thr-166 in the residue D*X*D*X*T, a haloacid dehalogenase motif that is specific for phosphatase activity. Similarly, CAL4 is phosphorylated at three different sites, Thr-39, Ser-75, and Ser-177, by STYK. Furthermore, the importance of OLE1(T166) phosphorylation site was demonstrated by using site directed mutation studies on T166 residue. Kinase assay clearly showed the lack of phosphorylation in the OLE1^T166A^ mutant by STYK.

Heterologous expression of plant OB protein oleosin in yeast cells has been shown to increase the cellular nonpolar lipid levels^8,22^. Therefore, we studied the effect OLE1 and its phosphorylation on the cellular lipid biosynthesis. The co-expression of OLE1 and STYK in yeast cells led to an increase in the cellular total lipid levels. The increase in the lipid levels was significantly higher than the cells overexpressing OLE1 alone. Interestingly, co-expression of OLE1 and STYK in yeast cells increased the lipid droplets. However, the effect was significantly reduced when STYK was co-expressed with OLE1^T166A.^ This further confirmed our *in vitro* observation that phosphorylation of OLE1 does play a role in modulating the lipid levels *in vivo*.

Most proteome studies on OBs were carried out on mature, dry seeds, presumably giving an impression of OBs as quiescent organelles. Therefore, in the current study, we used 36 h imbibed *A. thaliana* seeds with radicles. The mass spectrometric experiment revealed that the four distinctly separated phosphoproteins in the SDS-PAGE gel were identified as oleosin 1, oleosin 2, caleosin 4 and 12 S seed storage protein CRU3. In contrast, the major proteins that were identified in the 5^th^ band (clustered phosphoproteins) were polyubiquitin, 2 S seed storage protein 3 (AT2S3), 12 S seed storage protein CRU3, putative uncharacterized protein At2g40765 and a mitochondrial import receptor subunit TOM6 homolog. Previous studies have identified phosphorylation occurring in two of the identified proteins: oleosin 2 and CRU3. Specifically, a serine residue in the peptide “^13^HFQFQ(S)^**P**^PYEGGR^23^” from oleosin 2 is phosphorylated^24^. Further, a recent report demonstrated the phosphorylation of a proteolyzed fragment of oleosin 2^9^. Similarly, CRU3 is also phosphorylated at the residue Tyr-406 with the peptide “YNMNANEIL(Y)^**P**^CTGGQGR” (Meyer *et al*., 2012). 12 S seed storage proteins are the most abundant and phosphorylated proteins in *Arabidopsis* seeds. These studies further supported our proteomic approach and the phosphoprotein identification in OBs.

The *in vitro* and the microscopic evidences using yeast as a model system were further validated by assessing the OB phosphorylation status of the confirmed *styk* mutant. In accordance with our previous observation, the phosphorylation of most of the OB proteins was significantly reduced in the *styk* mutant as compared to wild-type OBs. This result further confirms STYK as a major protein kinase phosphorylating the OB proteins oleosin 1, oleosin 2, caleosin 4, and 12S seed storage protein CRU3. Interestingly, the microarray analyses of other STYK also supported by showing no significant alteration in its expression levels to compensate the *styk* (*At2g24360)* knock-out effect. We further studied the role of STYK mediated OB phosphorylation in *Arabidopsis* seed lipid accumulation. The seeds of the *styk* mutant accumulated 10–12% less TAG compared to WT, validating the role of STYK phosphorylation in cellular lipid accumulation.

The oilbody structural proteins plays an important role in regulating the morphology of oilbodies which in turn is correlated with the oil content and fatty acid compositions of seeds^23,25,26^. Studies in *B. napus* have shown that there exists a significant negative correlation between oilbody size and oil content^27^. Furthermore, *B. napus* with higher OB organelle to cell area ratio has been shown to have high oil content compared to the seeds with lower ratio. STYK by phosphorylating oleosin 1 could possibly prevent the coalescence of small oilbodies to form large droplets and increase the OB organelle to cell area ratio by dispersing them which ultimately increases the oil content. Furthermore, OLE1 has a “DXDXT” haloacid dehalogenase/phosphatase motif, STYK phosphorylation could modulate the enzyme activity to increase the seed oil content. Future studies would be directed to address how oleosin 1 phosphorylation affects the lipid levels in the Arabidopsis seeds.

In conclusion, the present study identified the different phosphorylated OB proteins of *Arabidopsis* and their corresponding protein kinase. The role of phosphorylation in the modulation of the structural function of oleosin 1, a major OB protein, is further discussed. Here, we also report the role of STYK mediated OB phosphorylation in affecting the seed oil content in *Arabidopsis*.

## Materials and Methods

### Materials

[γ-^32^P]ATP (3,000 Ci/mmol), [^32^P]orthophosphoric acid (3,000 Ci/mmol) and [^14^C]acetate (51 mCi/mmol) were obtained from Bhabha Atomic Research Centre (BARC), Mumbai, India. Phos-tag™ acrylamide was purchased from Wako Pure Chemical Industries, Ltd., Japan. Restriction endonucleases and *Pfu* polymerase were from New England Biolabs. The plasmid miniprep kit, agarose gel elution kit, PCR purification kit, nickel-nitrilotriacetic acid (Ni^2+^-NTA) matrix, and RNeasy plant minikit were purchased from Qiagen. HCS LipidTOX Red neutral lipid stain and BODIPY^®^ 493/503 (4,4-difluoro-1,3,5,7,8-pentamethyl-4-bora-3a,4a-diaza-s-indacene) were from Life Technologies. Casein kinase 2 was from New England Biolabs (#P6010S) Oligonucleotide primers, anti-His_6_ tag monoclonal antibody, and all other reagents were obtained from Sigma-Aldrich. An enhanced chemiluminescence kit (ECL) was obtained from Perkin Elmer. The At4g25140 (*OLE1*) clone, At1g70670 (*CAL4*) clone, Col-0 and *styk* knock-out lines were obtained from the *Arabidopsis* Biological Resource Center (ABRC). Yeast strains were purchased from the EUROSCARF collection center.

### STYK cloning and purification

GST recombinant fusion protein was purified using glutathione coupled to a Sepharose matrix (GE healthcare). The GST tag was removed using precession protease, and the protein was further purified using size-exclusion chromatography. Further, the protein was confirmed by immunoblot and used in the *in vitro* assays.

### Cloning and expression of OLE1 and CAL4

The coding sequences of the *OLE1* and *CAL4* gene in the pUNI vector were sub-cloned into the pRSET/C vector. The plasmid constructs used in the study are shown in Supplementary Table S2. The construct was then transformed into *E. coli*. The cells were induced with 0.5 mM isopropyl thio-ß-galactoside for 4 h at 37 °C to enable the expression of the gene. The cells were resuspended in a lysis buffer containing 50 mM Tris-HCl (pH 8.0), 300 mM NaCl, and protease inhibitor cocktail. The cells were lysed by mechanical disruption and the lysate was subjected to high-speed centrifugation (10,000 × g) for 20 min at 4 °C. Overexpression was confirmed by an anti-His_6_ tag monoclonal antibody. His-tagged recombinant OLE1 and CAL4 were extracted from the membranes using 6 M urea and 10 mM n-dodecyl-β-maltoside. The extracted protein was allowed to bind to Ni^2+^-NTA agarose beads, and the flow-through was collected. The column was washed with the lysis buffer containing 25 mM imidazole. The bound protein was eluted with 250 mM imidazole in the lysis buffer. The amount of purified recombinant protein was estimated using bovine serum albumin as a standard^28^. The fractions (1 mL each) were collected and analyzed on a 12% (w/v) SDS-PAGE. For immunoblot analysis, the purified protein was separated on a 12% SDS-PAGE and transferred to a Hybond ECL nitrocellulose membrane. Immunoreactive protein bands were detected by ECL using rabbit or mouse antisera as the primary antibodies and goat anti-rabbit or goat anti-mouse IgG, respectively, linked to peroxidase as the secondary antibodies. The purified protein was subjected to overnight dialysis and then used for the enzyme assay.

### Site-directed mutagenesis

OLE1 (T166A) was created using primer overlap extension method^29^. The PCR-amplified whole plasmid was treated with DpnI (10 units) for 1 h at 37 °C. The digested plasmid was transformed into DH5α competent cells. The mutations were confirmed by sequencing.

### In vitro kinase assay

The *in vitro* kinase assay mixture consisted of 50 mM Tris-HCl (pH 7.5), 10 mM MnCl_2_, 100 ng of STYK and 2 µg OLE1/CAL4 in the presence of 25 µM [γ-^32^P]ATP (3,000 dpm pmol^−1^), and the incubation was carried out at 30 °C for 30 min. The reaction was stopped by the addition of SDS-PAGE gel loading buffer. Phosphorylated products were separated by 15% (w/v) SDS-PAGE, and the labeled proteins were detected using a phosphorimager.

### Phosphoprotein enrichment

An *in vitro* kinase assay was performed using STYK as the enzyme and OLE1 as the substrate. The kinase assay mixture consisted of 50 mM Tris-HCl (pH 7.5), 10 mM MnCl_2_, 1 µg of STYK and 2 µg of OLE1 in the presence of 100 µM ATP, and the incubation was carried out at 30 °C for 30 min. The reaction was stopped by the addition of SDS-PAGE loading buffer. Phosphorylated products were separated by 10% (w/v) SDS-PAGE with 25 µM Phostag. The phosphorylated OLE1 proteins had a mobility shift that was detected in silver staining. Phosphorylated OLE1 was excised from the gel and subjected to in-gel trypsin digestion^30^.

### Mass spectrometric data acquisition

Digested peptides were reconstituted in 20 μL of 2% acetonitrile (ACN) with 0.1% formic acid, and 6 μL of the same was injected onto an Agilent zorbax SB300 C18 column (3.5 µm, 150 mm × 75 µm). The mobile phase consisted of buffer A (0.1% formic acid) and buffer B (80% acetonitrile 0.1% formic acid) (LCMS grade, Thermo fisher scientific). A linear gradient elution (11% buffer B to 100% buffer B) was done for 75 min to separate the phosphopeptides. The HPLC system was coupled online to a high-resolution LTQ Orbitrap mass spectrometer (Thermo Scientific). The mass spectrometer was operated in the data-dependent mode, in which a full-scan MS (from m/z 350–5000) was followed by 20 MS/MS scans of the most abundant ions using collision-induced dissociation (CID). Standard phosphopeptide mix (25 fmoles) spiked into a 250 fmol BSA digest was also analyzed to check the performance of the instrument. A minimum of two highly confident peptides were used as a prerequisite to identify the proteins.

The raw files obtained from LTQ-Orbitrap were searched directly against the *Arabidopsis* database that was downloaded from NCBI, with no redundant entries using the SEQUEST algorithm on Proteome Discoverer (Version 1.4; Thermo Fisher). The peptide precursor mass tolerance was set to 10 ppm, and the peptide fragment mass tolerance was set to 0.8 Da. Search criteria included a dynamic modification of +79.996 Da on normal phosphorylated serine, threonine, and tyrosine residues, +15.9949 Da on oxidized methionine and a static modification of +57.0214 Da on cysteine residues. Searches were performed with full tryptic digests and a maximum of two missed cleavages were allowed on peptides that were analyzed by the sequence database. False discovery rates (FDR) were set to 1% for each analysis. The number of unique phosphopeptides and non-phosphopeptides was manually counted and compared. Phosphorylation site localization from CID mass spectra was determined by PhosphoRS scores^31^. For phosphopeptides with inconclusive phosphorylation sites, the one with highest phosphoRS score was selected for further data interpretation.

### Yeast culture conditions

Yeast cells used in this study (Supplementary Table S3) were grown in YPD (1% (w/v) yeast extract, 2% (w/v) bacto peptone, 2% (w/v) glucose) or synthetic minimal media (SM) containing 0.67% yeast nitrogen base (Difco) that was supplemented with the appropriate amino acids and 2% (w/v) glucose or 2% (w/v) galactose. All of the experiments were conducted using cultures that were grown to the mid-logarithmic phase/stationary phase at 30 °C on a rotary shaker at 200 rpm.

### Cloning and heterologous expression of OLE1 and STYK in S. cerevisiae

The coding sequences of *OLE1*and *STYK* were sub-cloned in the yeast expression vectors pYES2-NT/C and p425-GPD, respectively. The plasmids containing *OLE1* and *STYK* were transformed into yeast cells by the lithium acetate method, and the transformants were selected on synthetic minimal media selection plates containing glucose^32^. The expression was confirmed by immunoblot using an anti-His_6_ tag monoclonal antibody. Yeast oil bodies were isolated using a published procedure^33^.

### Metabolic labeling of phospholipids and nonpolar lipids in yeast

The *OLE1*-His_6_-overexpressing yeast strain and vector control were precultured in 5 mL of synthetic minimal media without uracil and containing 2% (w/v) glucose. For *in vivo* labeling, cells at A_600_ 0.2 were transferred to fresh induction medium containing 2% (w/v) galactose and 0.2 µCi/mL [^14^C]acetate and grown for an additional 24 h until the cells reached the stationary phase. Cells (A_600_ = 25) were harvested by centrifugation, and lipids were extracted using chloroform/methanol (2:1; v/v). Individual phospholipids were separated by two-dimensional thin-layer chromatography on silica gel 60 using chloroform/methanol/25% ammonia (65:25:5, by vol.) as a first-dimension solvent and chloroform/methanol/acetone/acetic acid/water (50:10:20:15:5, by vol.) as the solvent for a second dimension^8^. Neutral lipids were separated by one-dimensional TLC using petroleum ether:diethylether:acetic acid (70:30:1, by vol.) as the solvent system. The labeled lipids were visualized by a phosphorimager. The spots corresponding to labeled lipids were scraped off to determine the radioactivity by liquid scintillation counting in a Microbeta 2 microplate counter (Perkin Elmer) using OptiPhase Supermix scintillation cocktail.

### Gas Chromatography

The total lipid content was extracted from yeast cells expressing the different constructs (OLE1, STYK, OLE1 + STYK). The extracted lipids were converted to total fatty acid methyl ester (FAMEs) and analyzed as described previously^34^.

### Yeast microscopic analysis

All the yeast microscopic pictures were captured by confocal microscopy using a Leica SP8 laser-scanning confocal microscope. The samples were viewed using a 100× oil immersion objective lens. For lipid droplet analysis, yeast cells were grown in synthetic media to stationary phase. The cells were washed with PBS and stained with BODIPY 493/503 (1 µg/mL) for 30 min at room temperature. Excess stain was removed by washing the cells with PBS. The cells were resuspended in 50% glycerol and viewed under a microscope. Comparison of lipid droplet in yeast cells overexpressing *OLE1* or vector control was performed using Huygens software. (Z section series projected to a 3D image) with the deconvoluted images from five different fields, with approximately 25 cells per field. The quantification of fluorescence was performed using image J software.

### Plant growth conditions

*Arabidopsis* wild-type (Col-0) and *styk* knock-out line (SALK_105195, SALK_120808 and SALK_144442c) plants were grown vertically on soil and on half-strength Murashige and Skoog medium (supplemented with 0.5% (w/v) sucrose and solidified with 0.7% agar) after 2 days of stratification at 4 °C. The plants were grown at 23 °C under a 16 h day (140 µmol m^−2^ sec^−1^) and 8 h night regime.

### Styk knock-out characterization

To identify the T-DNA insertions in the SALK_105195, SALK_120808, and SALK_144442 lines, the T-DNA borders of these SALK lines were amplified using the T-DNA left border and the corresponding gene-specific primers. Homozygous lines were identified by PCR using genomic DNA as a template from wild-type and SALK line leaves to confirm the disruption of the gene. The true knock-out lines were further confirmed by PCR using cDNA as a template. Ubiquitin was used as an internal control. RNA isolation was performed using a Qiagen RNeasy Plant minikit as per the manufacturer’s protocol with DNase treatment. The RNA concentration and purity were determined at an optical density ratio of 260/280 using the NanoDrop® ND-1000 spectrophotometer. cDNA was prepared using a high-capacity cDNA reverse transcription kit (Applied Biosystems).

### Isolation of oil bodies from Col-0 and styk knock-out mutant seeds

Oil bodies were isolated according to the method of Tzen *et al*.^4^, with the following modifications. Approximately 200 mg of mature seed was ground in 1 mL of ice-cold Buffer A (0.6 M sucrose, 50 mM Tris-HCl (pH 7.5), 10 mM KCl, 1 mM EDTA, 1 mM MgCl_2_, 5 mM β-mercaptoethanol and 1 mM phenylmethanesulfonyl fluoride (PMSF)) using a mortar and pestle. The crude homogenate was centrifuged at 1200 × g for 15 min to separate the unlysed cells and debris. The collected supernatant was layered with 1 mL of cold Buffer B (Buffer A containing 0.4 M sucrose instead of 0.6 M sucrose). The sample was centrifuged at 100,000 × g (Beckman Optima MAX-XP tabletop ultracentrifuge with TLS 55 rotor) for 30 min. The top hydrophobic layer was carefully removed using a spatula and resuspended in Buffer C (Buffer A with 2 M NaCl) using a 2 mL glass dounce homogenizer. The suspension was added to 1 mL of Buffer D (Buffer B with 2 M NaCl) and centrifuged at 100,000 × g for 30 min. The top layer was resuspended with a glass homogenizer in 1 mL of Buffer A, over layered with 1 mL of Buffer B and centrifuged at 100,000 × g for 30 min. The procedure was repeated, and the final oil body layer was resuspended in Buffer E (50 mM Tris-HCl (pH 7.5), 10 mM KCl, 1 mM EDTA, 1 mM MgCl_2_, 5 mM β-mercaptoethanol, and 1 mM PMSF).

### In vivo phosphorylation of A. thaliana oil body proteins

Col-0 and *styk* seeds were incubated in a microfuge tube containing MilliQ water and 500 µCi of [^32^P]orthophosphoric acid for 4 h and allowed to germinate on moistened paper (two layers of Whatman #2 filter paper soaked with 500 µCi of [^32^P]orthophosphoric acids in 5 mL of MilliQ water). The petri dishes were incubated at 23 °C in an 8-h-dark/16-h-light cycle for 36 h (the radical emerged from the seeds). The seeds were collected, washed with MilliQ water three times, and used to isolate oil bodies. The oil bodies were delipidated using diethyl ether, and the proteins were separated on a 15% SDS-PAGE. The phosphorylated proteins were identified by a phosphorimager (Typhoon FLA 9000). Phospho peptides from Col-0 OB proteins were identified as previously described.

### Microarray analyses of WT and styk knock-out lines

Briefly, radicle emerged seeds were used to isolate total RNA and 500 ng of total RNA from both WT and *styk 1* and 2 lines was used to prepare cDNA followed by cRNA generation. Whole genome micro array was performed as described previously. The microarray data was submitted to https://www.ncbi.nlm.nih.gov/geo/query/acc.cgi?acc=GSE94596 and assigned GSE94596 as the accession number. Gene expression profiling of all the STYK genes in Arabidopsis were analyzed using equal amount of total RNA and the amount of cDNA was normalized using endogenous actin primer and the expression profile was determined by using the respective STYK gene specific primers.

### Mass spectrometry analysis and quantification of seed triacylglycerol

Seed lipids from Col-0 and the *styk* mutant lines were extracted and analyzed as described in the Kansas Lipidomics Research Center (KLRC) protocol^35^. Ten plants of Col-0 and the *styk* lines were grown under controlled growth conditions. Seeds were pooled from ten plants and the lipid extraction was performed as four independent replicates for each plant line. Lipid extracts were dried completely and used for mass spectrometric analysis in the KLRC mass spectrometric facility. TAG analysis, was performed by continuous infusion into an ESI source on a triple quadrupole mass spectrometer (API14000, Applied Biosystems, USA). TAG molecular species were detected as [M + NH4]^+^ ions by a series of neutral loss scan targeted for C16-20 fatty acid losses. TAG data were produced by subtracting each background spectrum using ABI Analyst software and quantified using internal standards. Data obtained from four independent replicates for each line were averaged and were subjected to Student’s *t*-test at the *P* < 0.05 and P < 0.01 level to determine the statistical significance.

### Statistical Analysis

The experimental data are shown as the mean ± S.E., and data were analyzed using Student’s *t* test. Each experiment was repeated at least three times, and at least 125 cells were scored per experiment to quantify the microscopic data. Significance was determined at *p < 0.05; and **p < 0.01.

### Accession Numbers

Sequence data from this article can be found in the *Arabidopsis* Genome Initiative under the following accession numbers: OLE1 (At4g25140), corresponded to the S3 gene as reported in the Kim *et al*.^36^, CAL4 (At1g70670), and AtSTYK (At2g24360). *Arabidopsis* mutants used in this article can be found in the *Arabidopsis* Genome Initiative under the following accession numbers: styk1 (SALK_105195), styk2 (SALK_120808) and styk3 (SALK_144442). *styk* knock-out lines and WT microarray data deposited at the https://www.ncbi.nlm.nih.gov/geo/query/acc.cgi?acc=GSE94596, accession number: GSE94596.

### Data Availability

The author who is responsible for the distribution of materials that are integral to the findings that are presented in this article is Ram Rajasekharan (ram@cftri.com, director@cftri.res.in).

## Electronic supplementary material

Supplementary Information
