# Supplementary material for: Arabidopsis serine/threonine/tyrosine protein kinase phosphorylates oil body proteins that regulate oil content in the seeds
Source: Sci Rep. 2018 Jan 18;8:1154. doi: 10.1038/s41598-018-19311-3 (PMC5773694; doi:10.1038/s41598-018-19311-3)

Arabidopsis serine/threonine/tyrosine protein kinase phosphorylates oil body proteins that regulate oil content in the seeds

Iyappan Ramachandiran[1,3], Anitha Vijayakumar[2,3], Visvanathan Ramya[2], Ram Rajasekharan[1, 2 *]

[1] Department of Biochemistry, Indian Institute of Science, Bangalore 560012, India.

[2] Lipidomics Center, Central Food Technological Research Institute, Mysore, Karnataka 570020, India.

[3]These authors contributed equally to this article

Correspondence: Prof. Ram Rajasekharan, Lipidomics Center, Central Food Technological Research Institute, Mysore, Karnataka 570020, India

E-mail: ram@cftri.com, director@cftri.res.in, lipid@biochem.iisc.ernet.in

Supplementary Data

Supplementary Figure S1

A

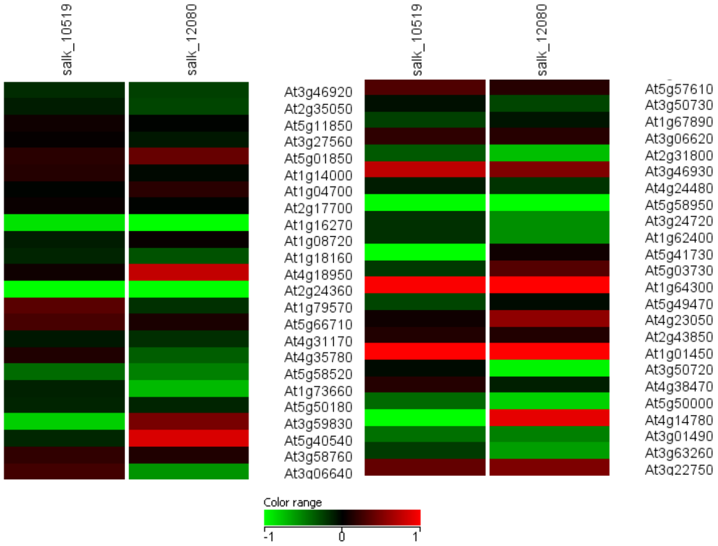

B

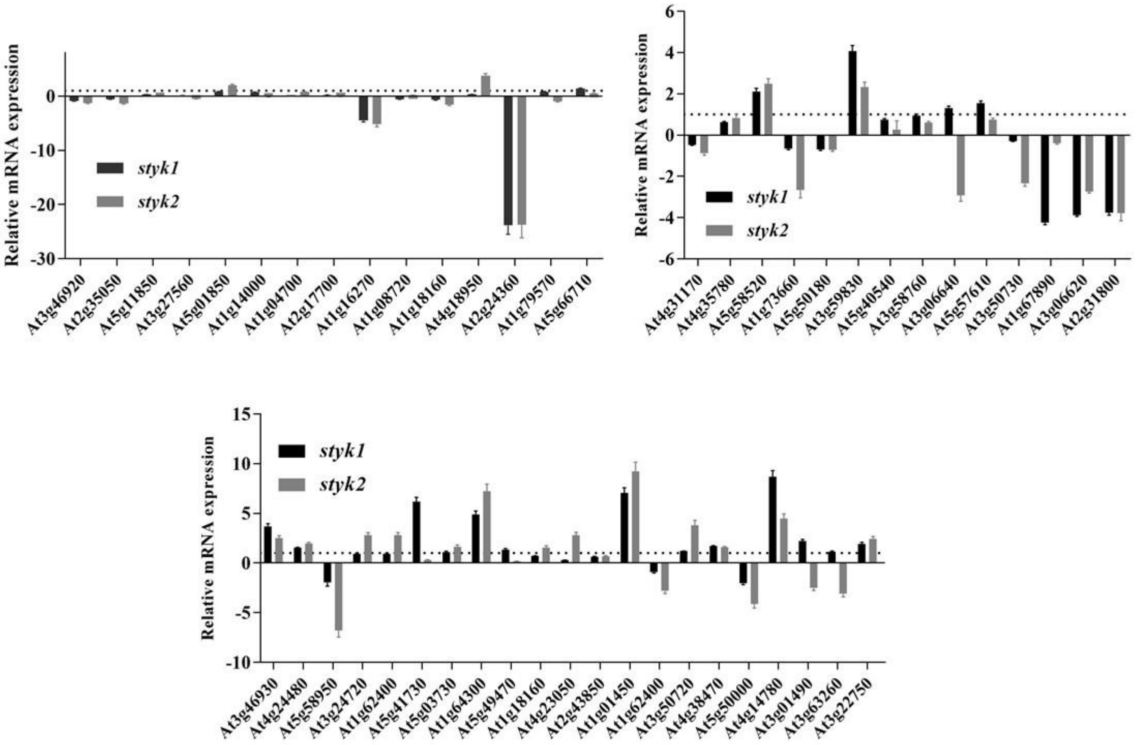

Supplementary Figure S1. Gene Expression profiling of Arabidopsis *STYK.* A, microarray expression profile of differentially expressed *Arabidopsis styk* knock-out lines. Scale bar represent the color change corresponding to the fold change as compared to wild-type **B**, qPCR analysis of STYK genes present in *Arabidopsis* using respective gene specific primers. Actin was employed as an internal control.

Supplementary Figure S2

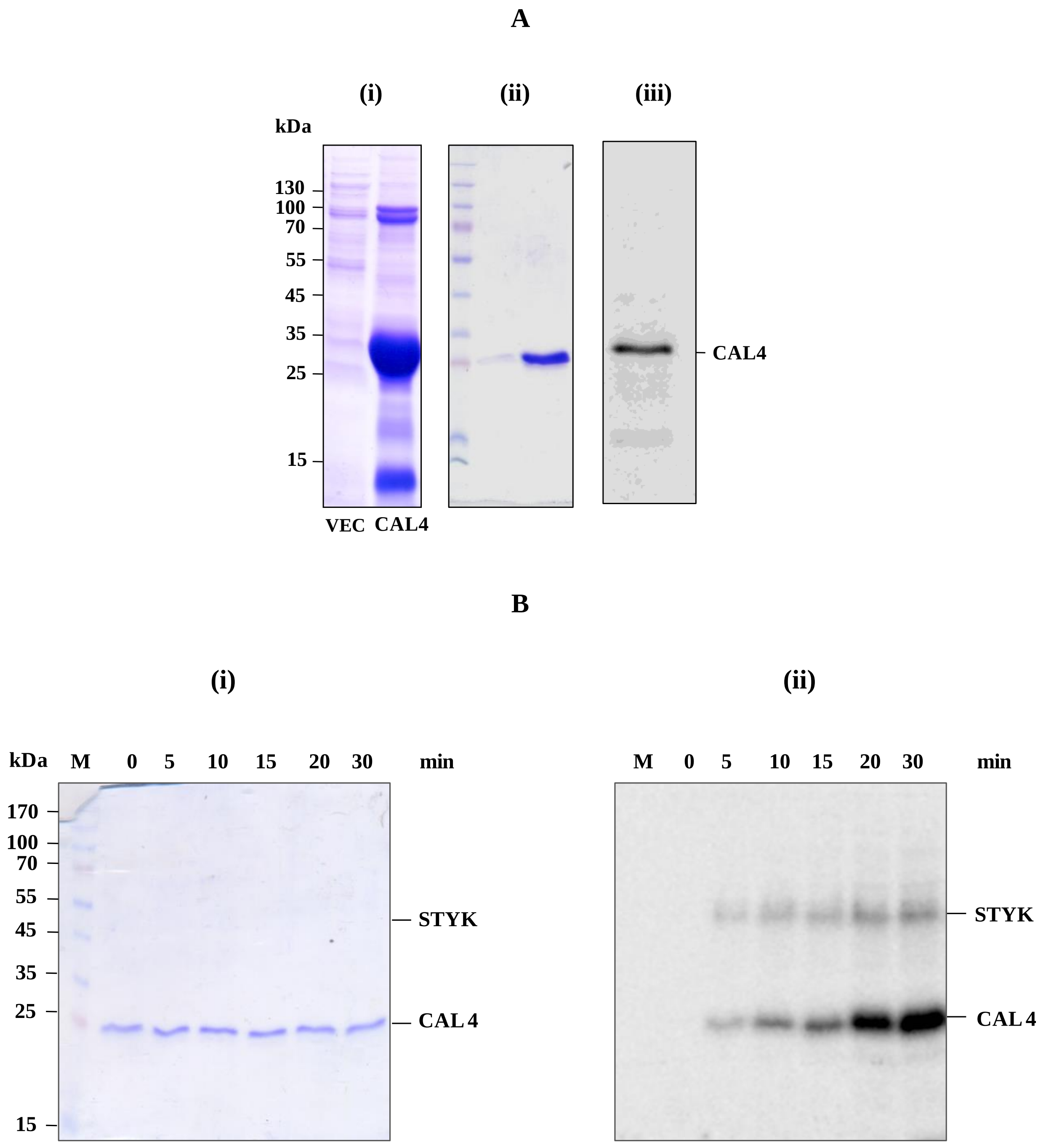

C

(i) **(ii)**

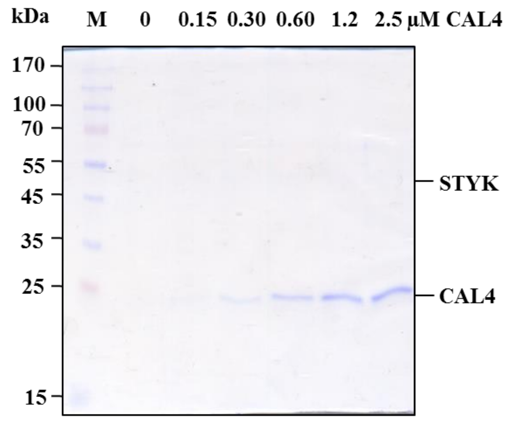

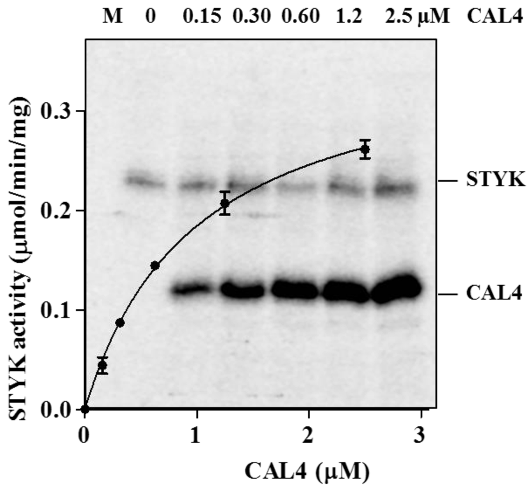

Supplementary Figure S2. AtSTY protein kinase phosphorylates CAL4. A, **(i)** SDS-PAGE profile of bacterially expressed CAL4. **(ii)** Ni^{2+}-NTA column purified CAL4. **(iii)** immunoblot analysis of CAL4 protein using anti-His_6 monoclonal antibody. *In vitro* phosphorylation of the purified, bacterially expressed CAL4 by STY protein kinase (STYK). **B**, time- and **C**,protein-dependent phosphorylation. After the reaction, the mixture was resolved with 15% SDS-PAGE and stained with (i) Coomassie brilliant blue stain followed by (ii) phosphorimaging. The radioactivity that was associated with the protein was determined by liquid scintillation counting. The data represent mean (±SD) of three independent experiments.

Supplementary Figure S3

A

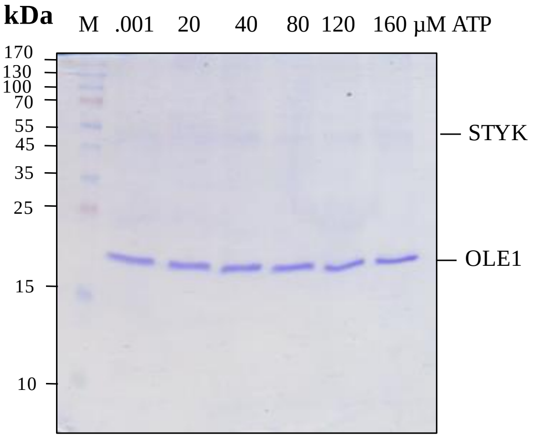

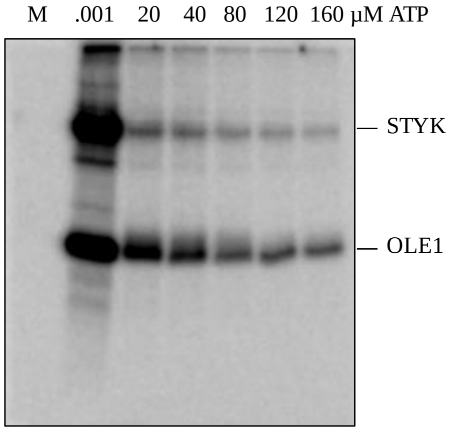

B

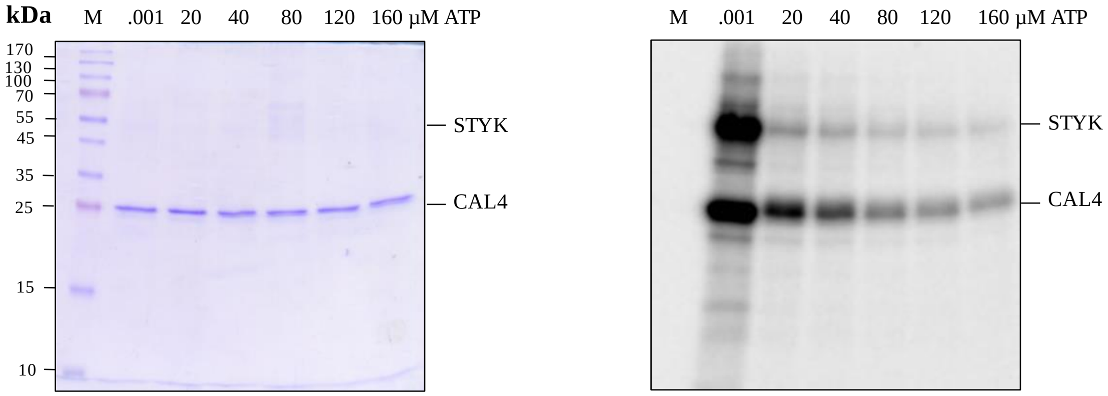

Supplementary Figure S3. ATP dependent phosphorylation of OLE1 and CAL4 by STYK. The *in vitro* kinase assay was performed using purified STYK and (**A**) OLE1 or (**B**) CAL4 with a constant [γ-^{32}P]ATP and an increasing concentration of cold ATP. The reaction mixture containing the phosphoproteins were resolved on a 15% SDS-PAGE and stained with Coomassie brilliant blue stain (CBB) followed by phosphorimaging.

Supplementary Figure S4

A **B** **C**

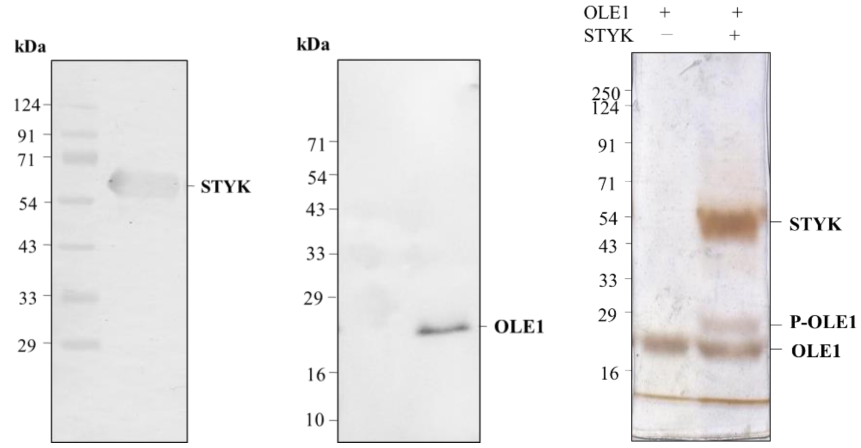

Supplementary Figure S4. Confirmation of OLE1, STYK and P-OLE1. Full length image of the immunoblot confirmation of purified A, STYK, B, OLE1. C, Phos-tag image of phosphorylated OLE1.

Supplementary Figure S5

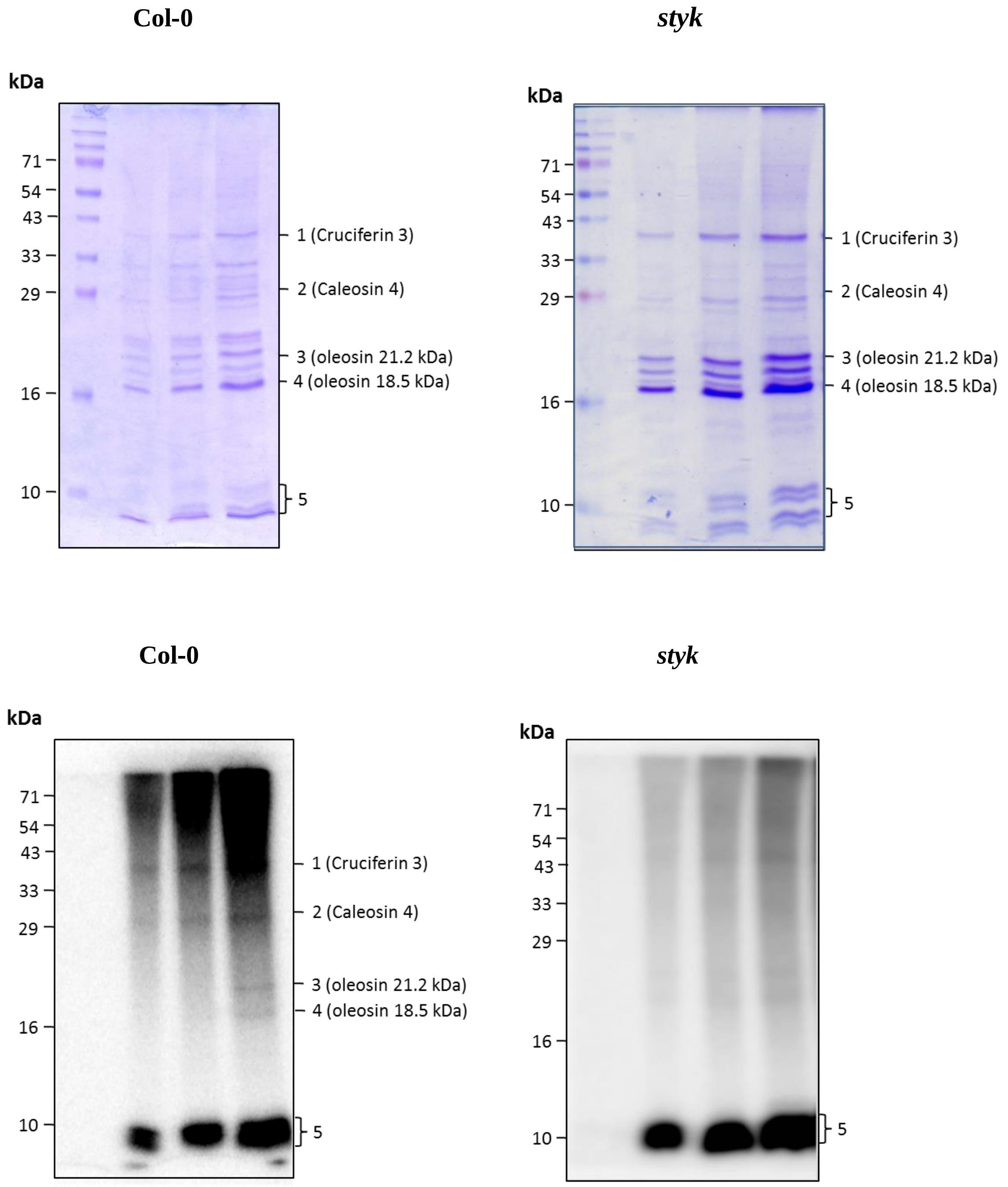

Supplementary Figure S5. STYK (At2g24360) phosphorylates Arabidopsis OB proteins. Col-0 and *styk* mutant seeds were imbibed in the presence of 250 µCi of [32P]orthophosphoric acid for 36 h (the radicles emerged). The seeds were ground, and the OBs were isolated and purified by sucrose density gradient centrifugation. The purified OBs were delipidated using diethyl ether, and the proteins were resolved on a 15% SDS-PAGE. Upper panel, Coomassie brilliant blue staining of proteins, Col-0 OB proteins (left), STYK mutant OB proteins (Right), Lower panel, Phosphorimaging of the respective gels.

Supplementary Figure S6

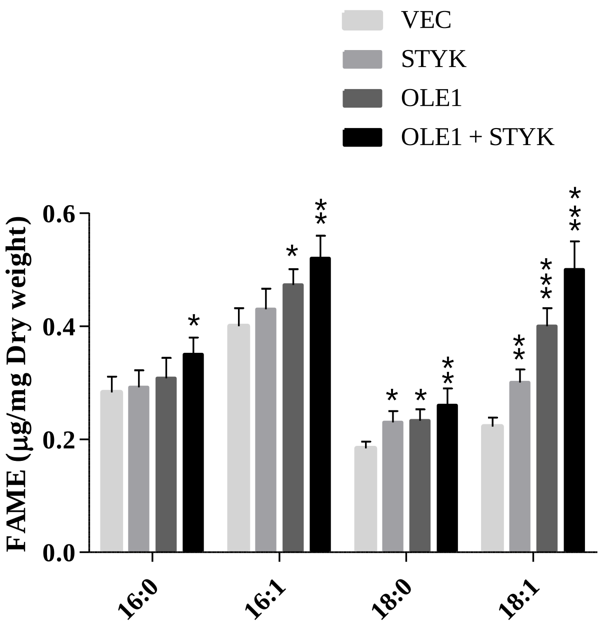

Supplementary Figure S6. Effect of OLE1 and STYK co-expression on yeast total lipid levels. The total lipids from the control yeast cells and yeast overexpressing either OLE1 or STYK or both were converted to fatty acid methyl esters (FAMEs). The FAMEs were injected to a DB5 column and analyzed by GC-MS. Heptadecanoic acid was used as an internal control. Error bars indicate SD ($n = 4$). Asterisks indicate significant differences compared to the vector controls (*$P < 0.05$, **$P < 0.01$, ***$P < 0.01$).

Supplementary Table 1

Identification of CAL4 phosphorylation site.LC-MS/MS identification of phosphoproteins resolved on a 12% SDS-PAGE and stained with Coomassie blue followed by phosphorimaging. *In vitro* kinase assay was performed with the purified CAL4 and STYK. The reaction mixture was passed through a Ni^{2+}-NTA affinity chromatography column to separate the CAL4 protein from free ATP, $MnCl_2$ and STYK. The eluted proteins were then separated on a 10% SDS-PAGE gel containing 25 µM Phostag acrylamide. The phosphoylated CAL4 was digested with trypsin, and the peptide mixture was analyzed by mass spectrometry.

Sequence	PSMs	Modifications	MH+ [Da]	Area	PhosphoRS Site Probabilities
**CAL4**					
^{172}AAYDG(S)^{P}LFEKLEK^{184}	2	S6(Phospho)	1550.71562	176561204	Y(3): 1.1; S(6): 98.9
^{29}NKDGIVYPSE(T)^{P}FQGFR^{44}	4	T11(Phospho)	1937.8751	122092506	Y(7): 0.0; S(9): 0.1; T(11): 99.9
^{31}DGIVYPSE(T)^{P}FQGFR^{44}	2	T9(Phospho)	1695.74468	76965348.3	Y(5): 0.0; S(7): 0.1; T(9): 99.9
^{73}GF(S)^{P}IWFPIEVK^{83}	1	S3(Phospho)	1402.68291	23517128.9	S(3): 100.0

Supplementary Table 2

Plasmids used in this study

Plasmids	Relevant characteristics	Source
pRSET-C	*E. coli* expression vector with His_6 tag fusion	Invitrogen
pGEX-6P-1	*E. coli* expression vector with GST tag fusion	Invitrogen
pYES2NT-C	Yeast expression vector with N-terminal His_6 tag fusion	Invitrogen
p425 GPD	Yeast expression vector with leucine selection	ATCC
pUG34-GFP	Yeast expression vector with GFP tag	(Ref)
pRSET-C-*OLE1*	*OLE1* coding sequence inserted into pRSET-C	This study
pRSET-C-*CAL4*	*CAL4* coding sequence inserted into pRSET-C	This study
pGEX-6P-1-*STYK*	*STYK* coding sequence inserted into pGEX-6P-1	This study
pYES2-NT/C-*OLE1*	*OLE1* coding sequence inserted into pYES2-NT/C	This study
p425 GPD-*OLE1*	*OLE1* coding sequence inserted into p425 GPD	This study
p425 GPD-*STYK*	*STYK* coding sequence inserted into p425 GPD	This study

Supplementary Table 3

Strains used in this study

Strain	Genotype	Source
Yeast		
BY4741	*Mat a; his3Δ1; leu2Δ0; met15Δ0; ura3Δ0*<br>*E. coli* expression vector with GST tag fusion	Euroscarf collection, Frankfurt, Germany
W303-1A	*MATa leu2-3,112 trp1-1 can1-100 ura3-1 ade2-1 his3-11,15 ybp1-1*	Kind gift of Prof. Joel M. Goodman (Adeyo *et al.*, 2011)
E.coli		
DH5α	F^- ϕ80d*lacZ*ΔM15 Δ(*lacZYA-argF*)U169 *deoR recA1endA1 hsdR17*(r_k^- m_k^+) *phoA supE44* λ^- *thi-1 gyrA96 relA1*	Invitrogen
BL21(DE3)pLysS	F^- *ompT hsdSB* ($r_B^-m_B^-$) *gal dcm* (DE3) pLysS	Invitrogen

Original blots and phosphor-image:

Figure 1

A

Left panel

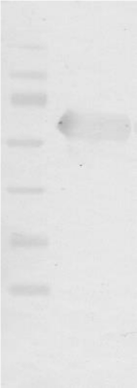

Right panel

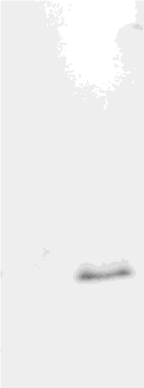

B (ii)

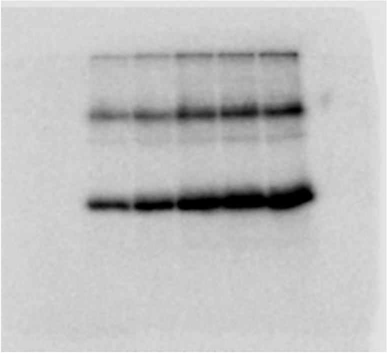

C (ii)

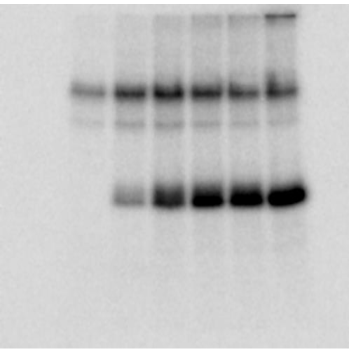

Figure 2

A (ii)

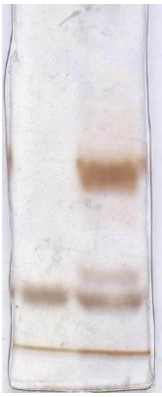

B

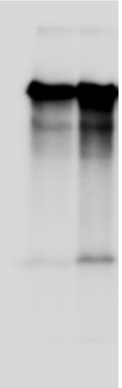

Figure 4

Right panel

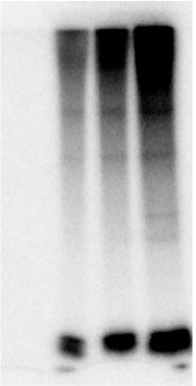

Figure 5

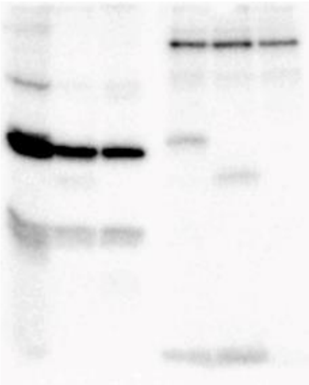

Figure 6D

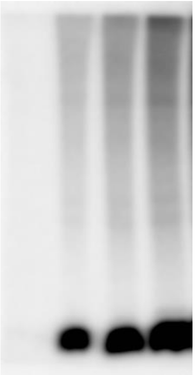

Supplementary Figure S2

A (iii)

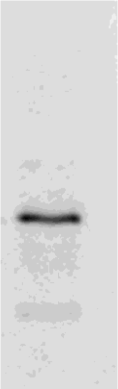

B (ii)

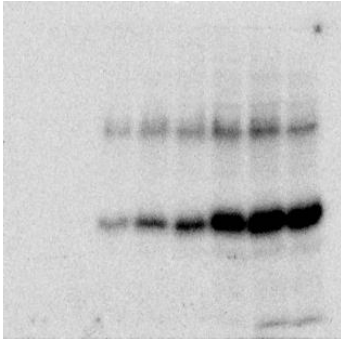

C (ii)

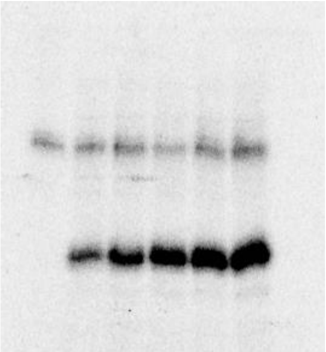

Supplementary Figure S3

A

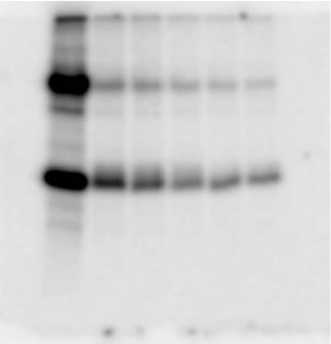

B

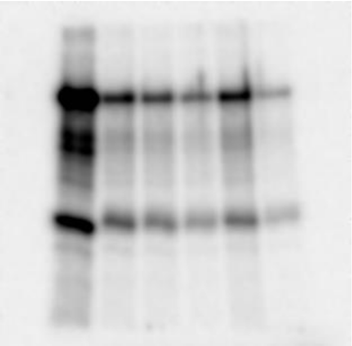

Supplement: Supplementary file 1 — Supplementary Information [file 41598_2018_19311_MOESM1_ESM.pdf]
